# Supplementary material for: FcRn-silencing of IL-12Fc prevents toxicity of local IL-12 therapy and prolongs survival in experimental glioblastoma
Source: Nat Commun. 2025 May 22;16:4751. doi: 10.1038/s41467-025-59971-0 (PMC12098678; doi:10.1038/s41467-025-59971-0)
Supplement: Supplementary file 1 — Supplementary Information [file 41467_2025_59971_MOESM1_ESM.pdf]

Supplemental Information

FcRn-silencing of IL-12Fc prevents toxicity of local IL-12 therapy and prolongs survival in experimental glioblastoma

Michal Beffinger[1,2,13], Linda Schellhammer[1,13], Betül Taskoparan[1,13], Sereina Deplazes[1,2], Ulisse Salazar[1], Nazanin Tatari[3], Frauke Seehusen[4], Leopold von Balthazar[5], Carl Philipp Zinner[1,6], Sabine Spath[2], Tala Shekarian[3], Marie-Françoise Ritz[3], Marta McDaid[3], Pascal Egloff[7], Iwan Zimmermann[7,12], Hideho Okada[8,9], E. Sally Ward[10], Jack Rohrer[5], Markus A. Seeger[7], Thorsten Buch[1], Gregor Hutter[3,11], Johannes vom Berg[1,2]

1 Institute of Laboratory Animal Science, University of Zurich, Schlieren, Switzerland

2 InCephalo AG, Allschwil, Switzerland

3 Brain Tumor Immunotherapy and Biology Lab, Department of Biomedicine, University Hospital and University of Basel, Basel, Switzerland

4 Laboratory for Animal Model Pathology (LAMP), Institute of Veterinary Pathology, University of Zurich, Zurich, Switzerland

5 Institute of Chemistry and Biotechnology, Zurich University of Applied Sciences, Waedenswil, Switzerland

6 Institute of Medical Genetics and Pathology, University Hospital Basel, Basel, Switzerland

7 Institute of Medical Microbiology, University of Zurich, Zurich, Switzerland

8 Department of Neurological Surgery, University of California, San Francisco, California, USA

9 Parker Institute for Cancer Immunotherapy, San Francisco, California, USA

10 Cancer Sciences Unit, Centre for Cancer Immunology, University of Southampton, Southampton, UK

11 Department of Neurosurgery, University Hospital of Basel, Basel, Switzerland

12 Present address: Linkster Therapeutics AG, Zurich, Switzerland

13 These authors contributed equally: Michal Beffinger, Linda Schellhammer, Betül Taskoparan

Corresponding Author: Johannes vom Berg

Email: johannes.vomberg@uzh.ch

Contents:

Figures S1 to S11
Tables S1 to S3
Supplemental References

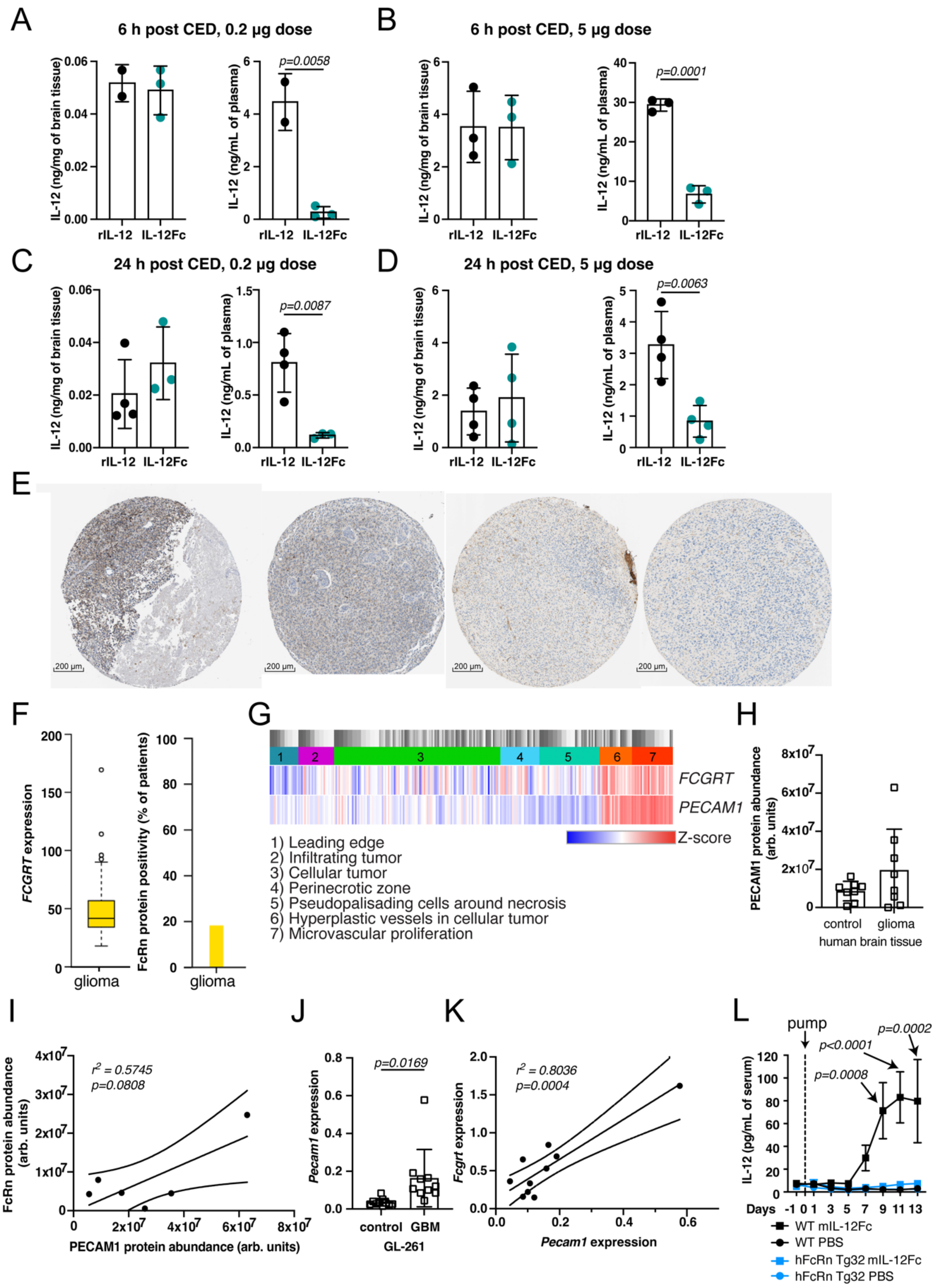

Figure S1. FcRn is more abundant in brain cancer and limits exclusive localization of IL-12Fc to the brain. (A-D) 0.2 or 5 µg of IL-12 or IL-12Fc were administered into the striatum of hFcRn Tg32 mice via CED. IL-12 levels in the ipsilateral brain hemisphere and in plasma 6 h (A, B) and 24 h (C, D) after injection. Unpaired

two-tailed t-test., Mean ± SD. rIL-12 6 h 0.2 µg: n=2, IL-12Fc 6 h 0.2 µg: n=3, rIL-12 6 h 5 µg: n=3, IL-12Fc 6 h 5 µg: n=3, rIL-12 24 h 0.2 µg: n=4, IL-12Fc 24 h 0.2 µg: n=3, rIL-12 24 h 5 µg: n=4, IL-12Fc 24 h 5 µg: n=4 animals. **(E)** Selected examples of cellular and vascular FcRn immunohistochemistry staining in glioma specimens. Human Protein Atlas, Image credit: proteinatlas.org. Antibodies HPA015130 and HPA012122, accessed on 28.01.2025 [84]. **(F)** Detectable transcript of FcRn (fragments per kilobase transcript per million, left) and % patients with detected FcRn protein (histology, right). Median and 25th and 75th percentile with points displayed as outliers if they are above or below 1.5x interquartile range. Data set of 153 glioma cases, data source as in E. **(G)** Levels of *FCGRT* (FcRn) and *PECAM1* (CD31) gene transcripts (z-score) in different anatomic regions of glioblastoma tissue. Data set of 122 RNA-Seq samples from ten tumors. Data from Ivy Glioblastoma Atlas Project, accessed 28.01.2025 [85]. **(H)** PECAM1 protein abundance in brain tissue from patients with non-related disease (epilepsy) vs glioma according to dataset from [20]. Mean ± SD. **(I)** Correlation of protein abundance PECAM1 vs FcRn in human brain tissue of non-related disease (epilepsy) vs glioma according to dataset from [20]. Curved lines indicate the 95% confidence interval for the regression linear line. Statistical analysis for deviation from zero. **(J)** Relative expression of *Pecam1* in GL-261 tumor-bearing hemisphere (day 21 post implantation) and healthy contralateral hemisphere was measured by RT-qPCR, plotted expression fold change $2^{-\Delta Ct}$ to reference gene *Hprt.* Paired two-tailed t-test. Mean ± SD. n=10 animals/group. **(K)** Correlation of *Fcgrt* and *Pecam1* gene expression (fold change $2^{-\Delta Ct}$ to *Hprt* control) in GL-261 tumor-bearing hemisphere (day 21 post implantation). Curved lines indicate the 95% confidence interval for the linear regression line. Statistical analysis for deviation from zero. n=10 animals. **(L)** Continuous i.t. delivery of murine IL-12Fc (mIL-12Fc; squares) or PBS (circles) over 14 days by osmotic minipumps into GL-261 brain-tumor-bearing C57BL/6 (WT, n=4 animals/group, black) or hFcRn Tg32 (n=5 animals/group, blue) mice. Serum IL-12 levels were measured by multiplex bead assay in reference to days post pump implantation. Two-way ANOVA with Sidak's multiple comparison test mIL-12Fc in C57BL/6 vs hFcRn Tg32 mice. Mean ± SEM. Source data are provided as a Source Data file.

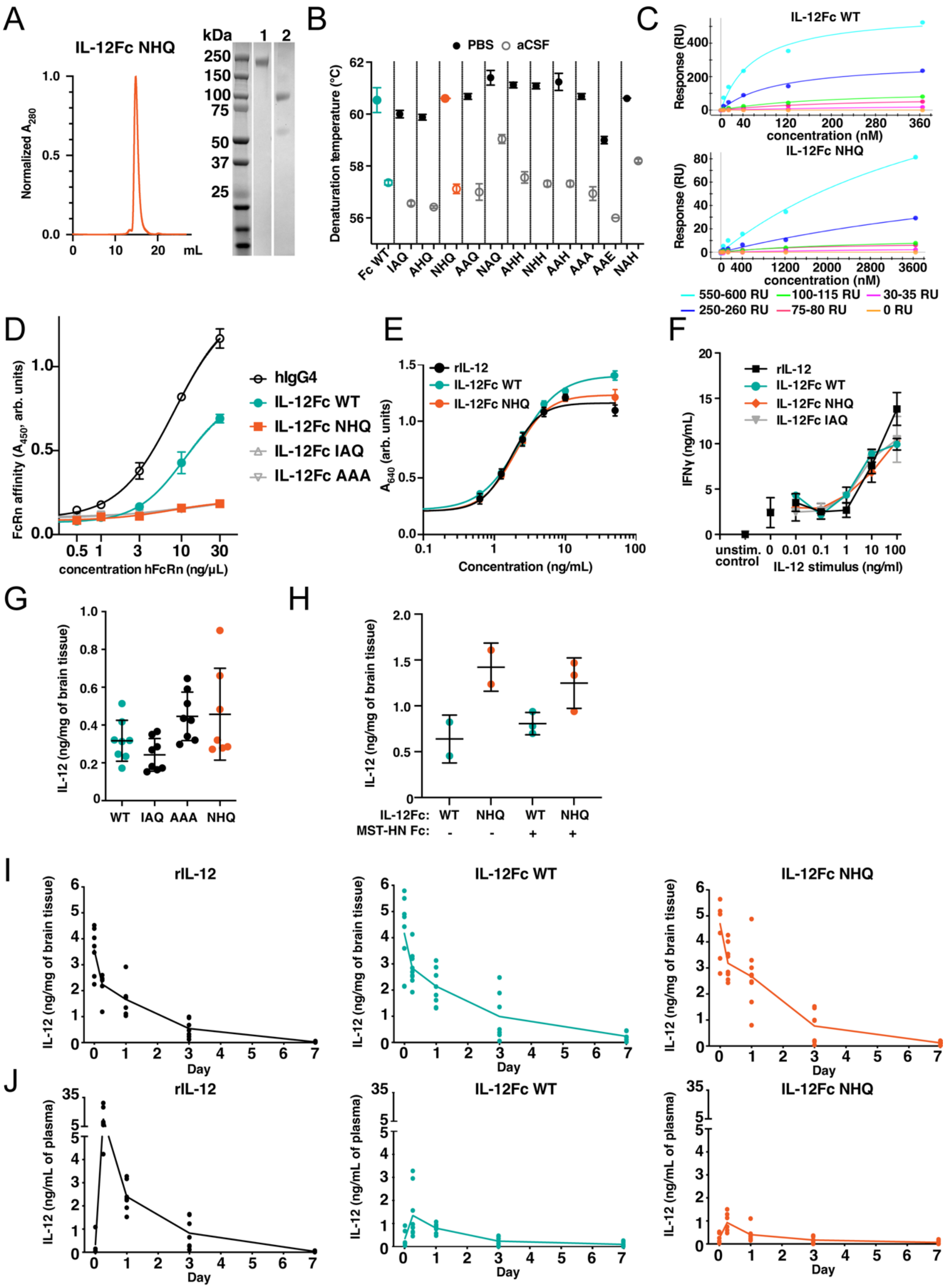

Figure S2. Characterization of IL-12Fc variants with low FcRn affinity. (A) Size exclusion chromatogram (left, normalized absorbance at 280 nm; A_{280}) and SDS-PAGE (right) of hIL-12Fc NHQ protein under native (lane 1) and reducing conditions (lane 2). **(B)** Thermal shift assay in PBS (filled points) and artificial

cerebrospinal fluid (aCSF; empty points). Denaturation temperature; Mean ± SD. **(C)** SPR analysis: Equilibrium analysis of the data shown in Figure 2 B including all FcRn coating densities. Lanes coated with 550-600 response units (RU, light blue), 250-260 RU (dark blue), 100-115 RU (green), 75-80 RU (red), 30.35 RU (pink), 0 RU (orange). **(D)** Binding to hFcRn measured by an ELISA-based FcRn binding assay at pH=6.0, immobilizing IL-12 and detecting bound FcRn. hIgG4 (black), IL-12Fc WT (green), IL-12Fc NHQ (orange), IL-12Fc IAQ (gray) and IL-12Fc AAA (gray). Absorbance at 450 nm (A_{450}). Mean ± SD. **(E)** IL-12R engagement of indicated variants on HEK-Blue IL-12 reporter cell line measured by a colorimetric readout (absorbance at 640 nm; A_{640}). Representative experiment for IL-12Fc NHQ vs WT variant vs rIL-12 is shown; complete data in Table S1. IL-12Fc WT (green), IL-12Fc NHQ (orange), rIL-12 (black). Mean ± SD. **(F)** Human PBMCs were stimulated with anti-CD3 100 ng/mL with or without IL-12 stimulus in varying concentrations for 24 h. IFNγ in the supernatant was quantified by ELISA. Mean ± SD, one out of three donors shown. IL-12Fc WT (green), IL-12Fc NHQ (orange), IL-12Fc IAQ (gray), rIL-12 (black). **(G)** IL-12 levels in the ipsilateral hemisphere 24 h after intraparenchymal administration of 1 µg of indicated IL-12Fc variants via CED into hFcRn Tg32 mice. Mean ± SD. IL-12Fc WT: n=8, IL-12Fc IAQ: n=8, IL-12Fc AAA: n=8, IL-12Fc NHQ: n=7 animals. **(H)** IL-12 levels in the ipsilateral hemisphere 6 h after intraparenchymal administration of 1 µg of indicated IL-12Fc variants with or without 10 µg of FcRn inhibitor MST-HN Fc via CED in hFcRn Tg32 mice. Mean ± SD. IL-12Fc WT: n=2, IL-12Fc NHQ: n=2, IL-12Fc WT + MST-HN Fc: n=3, IL-12Fc NHQ + MST-HN Fc: n=3 animals. **(I, J)** Extended PK analysis. IL-12 concentration in the ipsilateral hemisphere (I) and in the plasma (J) after intraparenchymal administration of 1 µg of indicated IL-12Fc variants via CED into hFcRn Tg32 mice as described in figure 2 E. Mean and individual data points (n=7-12 / timepoint / group). Outlier removal based on Grubbs' test. Source data are provided as a Source Data file.

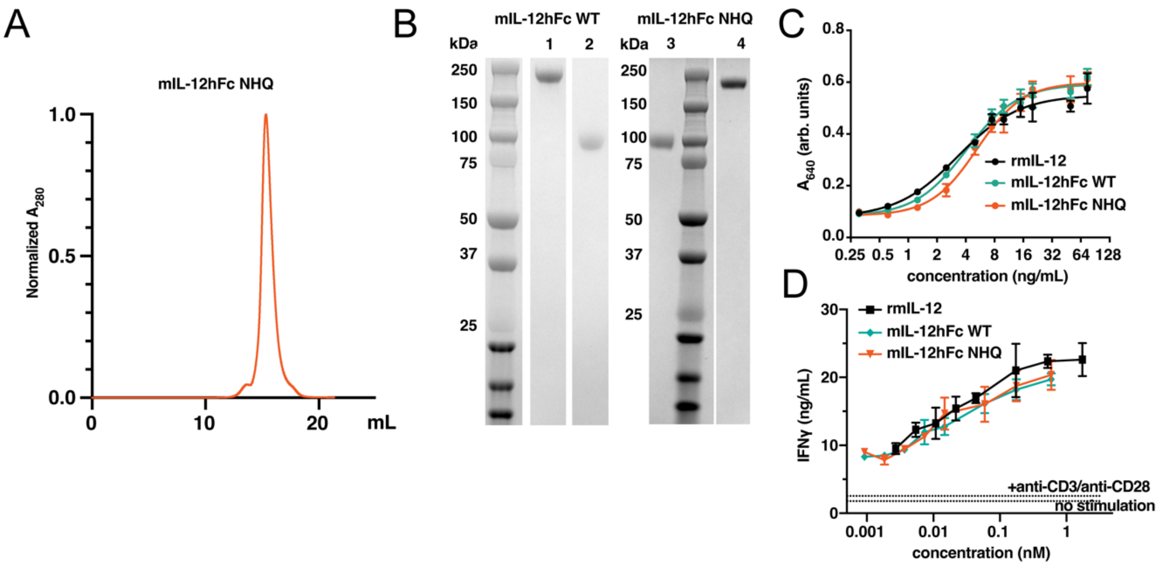

Figure S3. Characterization of mIL-12hFc variants. (A) Size-exclusion chromatography and **(B)** SDS-PAGE of mIL-12hFc WT native (lane 1), reduced (lane 2) and mIL-12hFc NHQ native (lane 3) and reduced (lane 4). Lanes cropped from corresponding gels. Normalized absorbance at 280 nm (A_{280}). **(C)** Bioactivity of mIL-12hFc on HEK-Blue reporter assay as in Fig. S2E. Absorbance at 640 nm (A_{640}) Mean ± SD. mIL-12hFc WT (green), mIL-12hFc NHQ (orange), rmIL-12 (black). **(D)** Bioactivity of mIL-12hFc variants or rmIL-12 on C57BL/6 splenocytes in the presence of agonistic anti-CD3/CD28 antibody. Mean ± SD. mIL-12hFc WT (green), mIL-12hFc NHQ (orange), rmIL-12 (black). Source data are provided as a Source Data file.

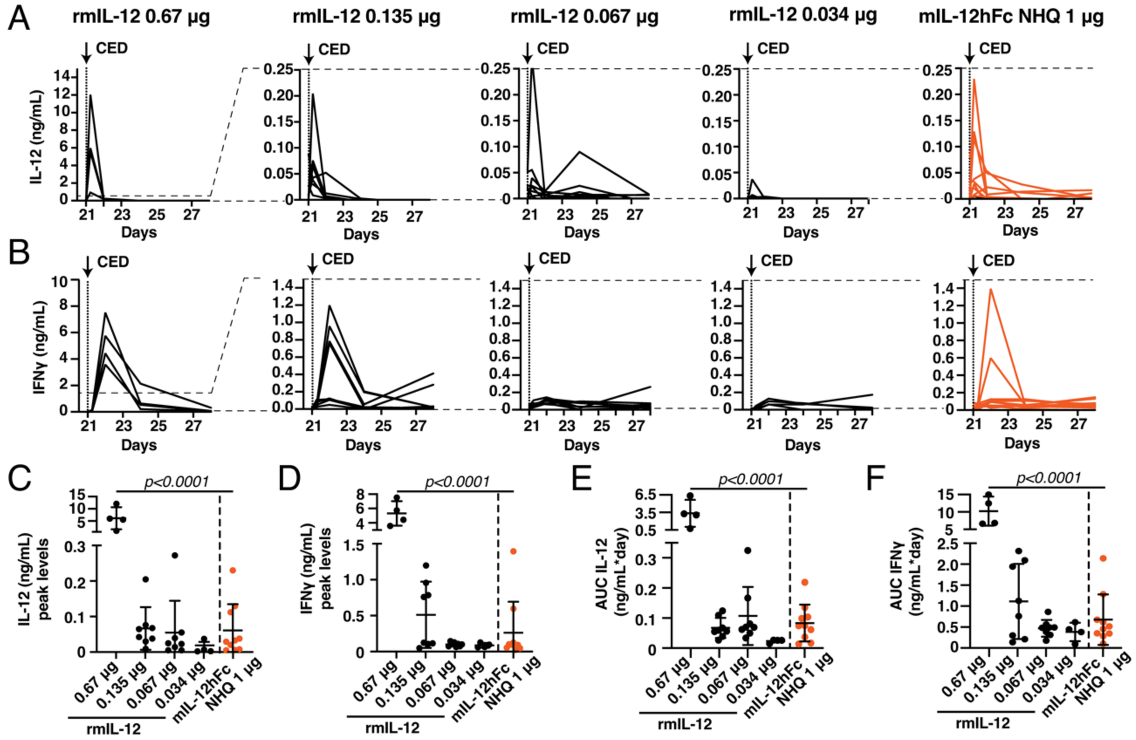

Figure S4. Reduced systemic prevalence of IL-12Fc NHQ enables higher dosing compared to IL-12. (A-B) Systemic exposure upon different i.t. doses of recombinant IL-12 (0.67 µg rmIL-12 corresponding to 1 µg of mIL-12hFc NHQ, equimolar amount adjusted to IL-12 stoichiometry, doses indicated above graphs): GL-261:luc tumor-bearing mice were treated as in Figure 3 A and plasma cytokine levels were measured as in Figure 3 E and H. IL-12 **(A)** and IFNγ **(B)** levels in plasma. Data pooled from two independent experiments. Days post tumor injection. Dotted vertical lines indicate CED. Dashed horizontal lines depict the change in the axis scale between the plots for rmIL-12 0.67 µg and other experimental cohorts. rmIL-12 0.67 µg: n=4, rmIL-12 0.135 µg: n=8, rmIL-12 0.067 µg: n=8, rmIL-12 0.034 µg: n=4, mIL-12hFc NHQ 1 µg: n=10 animals. **(C, D)** Peak values of plasma cytokine levels from **(C)** data in (A): IL-12 (6 h post CED) and **(D)** data in (B): IFNγ (24 h post CED). Mean ± SD. One-way ANOVA with Dunnett's test vs mIL-12hFc NHQ. **(E, F)** Overall exposure (total area under the curve to baseline, AUC), **(E)** data from (A) IL-12 or **(F)** data from (B) IFNγ. Mean ± SD. Statistical analysis as in C and D. Source data are provided as a Source Data file.

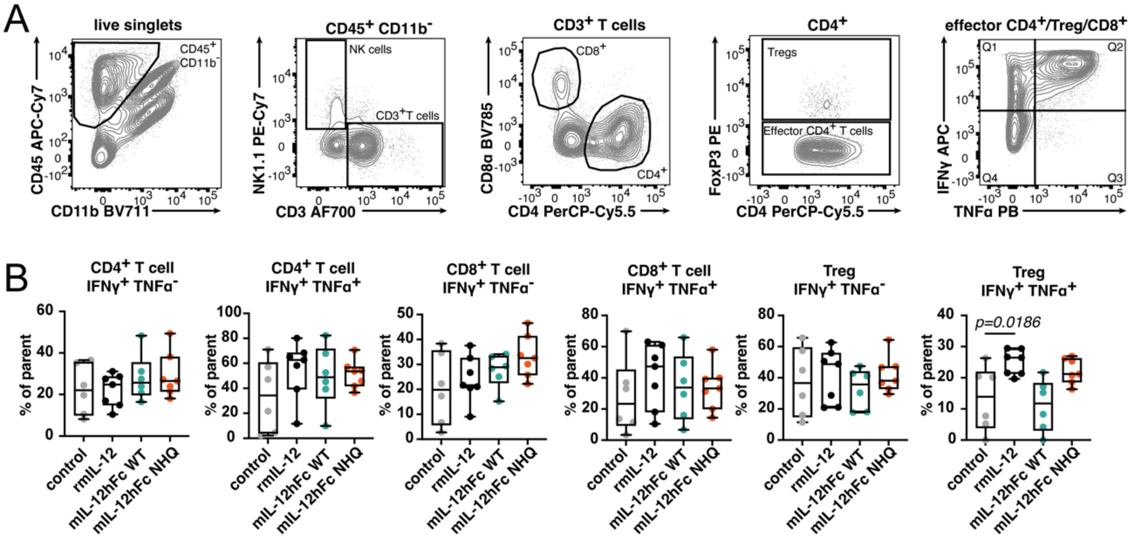

Figure S5. T cell repolarization within IL-12-treated tumors. (A, B) T cell infiltrate at day 28, seven days after the first i.t. IL-12 dose. Animals from experiments described in Figure 3 A and C. **(A)** Flow cytometry gating strategy for the identification and characterization of T cells in GL-261 tumors. Live singlets were selected, followed by gating on $CD45^+$ $CD11b^-$ cells to exclude non-immune, microglia and myeloid cells, respectively. From the $CD45^+$ $CD11b^-$ population, NK cells and $CD3^+$ T cells were identified. The $CD3^+$ T cell population was further divided into $CD4^+$ and $CD8^+$ T cells. Within the $CD4^+$ T cell population, effector $CD4^+$ T cells and regulatory T cells (Tregs) were distinguished based on Foxp3 expression. Effector $CD4^+$ T cells, Tregs, and $CD8^+$ T cells were assessed for frequencies of TNFα and IFNγ. **(B)** Frequency of effector $CD4^+$ T cells, $CD8^+$ T cells and Tregs producing only IFNγ or IFNγ and TNFα within the parent cell population. Whiskers represent the minimum to maximum, middle line represents the mean. One-way ANOVA with Dunnett's test to control group. Control: n=6, rmIL-12: n=7, mIL-12hFc WT: n= 6, mIL-12hFc NHQ: n=7 animals. Source data are provided as a Source Data file.

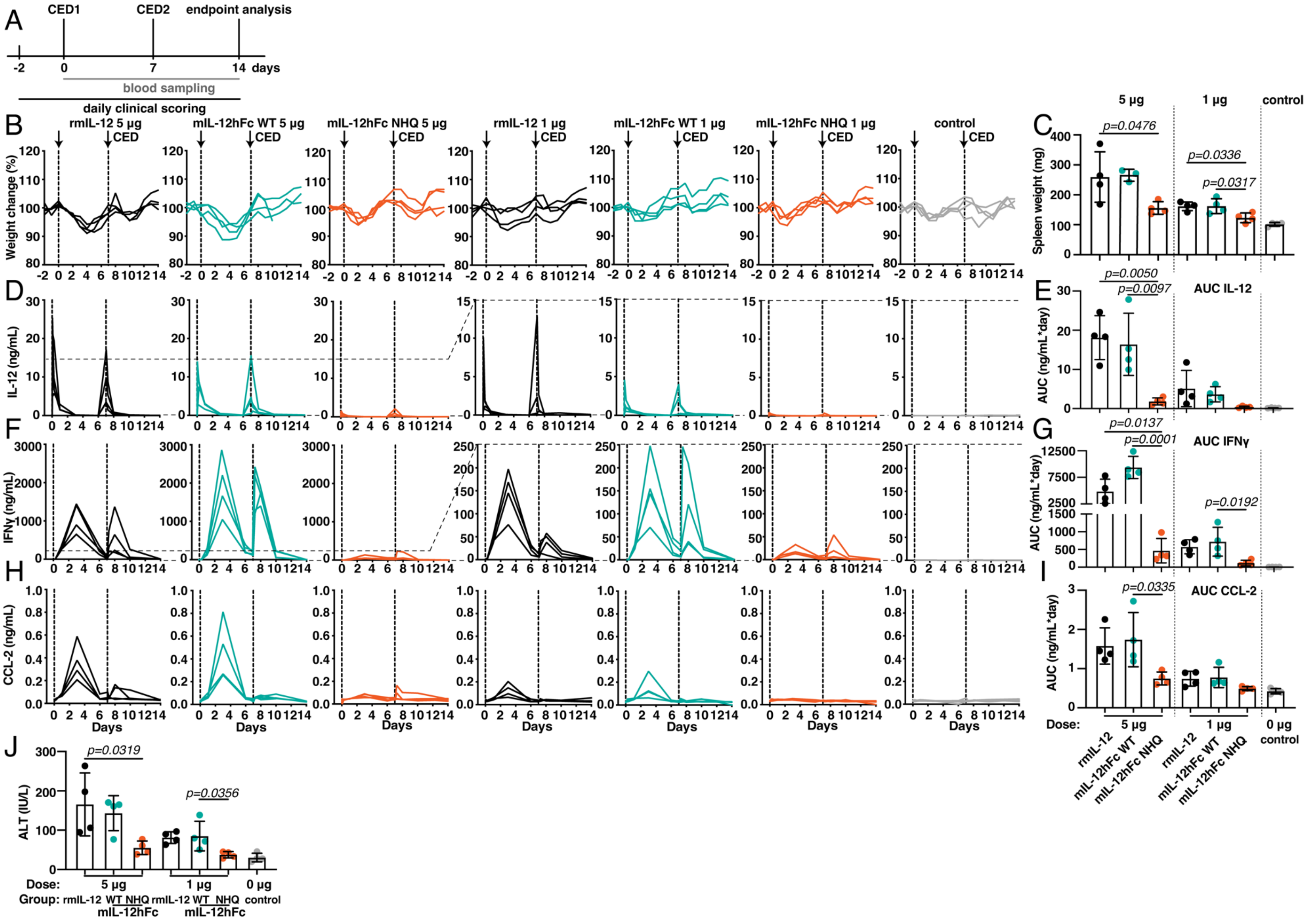
A
CED1
CED2
endpoint analysis
-2
0
7
14 days
blood sampling
daily clinical scoring
B
rmIL-12 5 µg
mIL-12hFc WT 5 µg
mIL-12hFc NHQ 5 µg
rmIL-12 1 µg
mIL-12hFc WT 1 µg
mIL-12hFc NHQ 1 µg
control
CED
Weight change (%)
D
IL-12 (ng/mL)
F
IFNγ (ng/mL)
H
CCL-2 (ng/mL)
Days
C
5 µg
1 µg
control
Spleen weight (mg)
p=0.0476
p=0.0336
p=0.0317
E
AUC IL-12
AUC (ng/mL*day)
p=0.0050
p=0.0097
G
AUC IFNγ
p=0.0137
p=0.0001
p=0.0192
I
AUC CCL-2
p=0.0335
Dose:
0 µg
rmIL-12
mIL-12hFc WT
mIL-12hFc NHQ
J
ALT (IU/L)
p=0.0319
p=0.0356
Group:
WT
NHQ
mIL-12hFc

Figure S6. C3H/HeJ animals can tolerate high intracranial doses of FcRn silenced mIL-12hFc NHQ, while rmIL-12 and mIL-12hFc WT elicit systemic toxicity. (A) C3H/HeJ animals received two doses of mIL-12hFc NHQ, rmIL-12 or mIL-12hFc WT (1 µg or 5 µg) on days 0 and 7 by intracranial injection via CED. **(B)** Weight change of individual animals per cohort, normalized to average weight from the day -2 to 0. **(C)** Spleen weight. Data from animals that reached withdrawal criteria before the planned endpoint were excluded, as per the recommendations of the Society of Toxicologic Pathology [86]. One-way ANOVA with Dunnett's test mIL-12hFc NHQ to mIL-12hFc WT and rmIL-12 group per dose level. Mean ± SD. Plasma concentrations of IL-12 **(D, E)**, IFNγ **(F, G)** and CCL-2, a soluble marker for liver damage [87] **(H, I)** data from individual animals assessed by multiplex bead assay. Vertical lines indicate CED. Dashed horizontal lines depict the change in the axis scale between the plots for rmIL-12 5 µg and other experimental cohorts. Overall exposure (AUC) analyzed by one-way ANOVA with Dunnett's test mIL-12hFc NHQ to rmIL-1 hFc WT and rmIL-12 groups per dose level. **(J)** Alanine transaminase (ALT) plasma activity on day 3. One-way ANOVA with Dunnett's test mIL-12hFc NHQ to mIL-12hFc WT and rmIL-12 groups per dose level. Mean ± SD. n=4 animals/group. Source data are provided as a Source Data file.

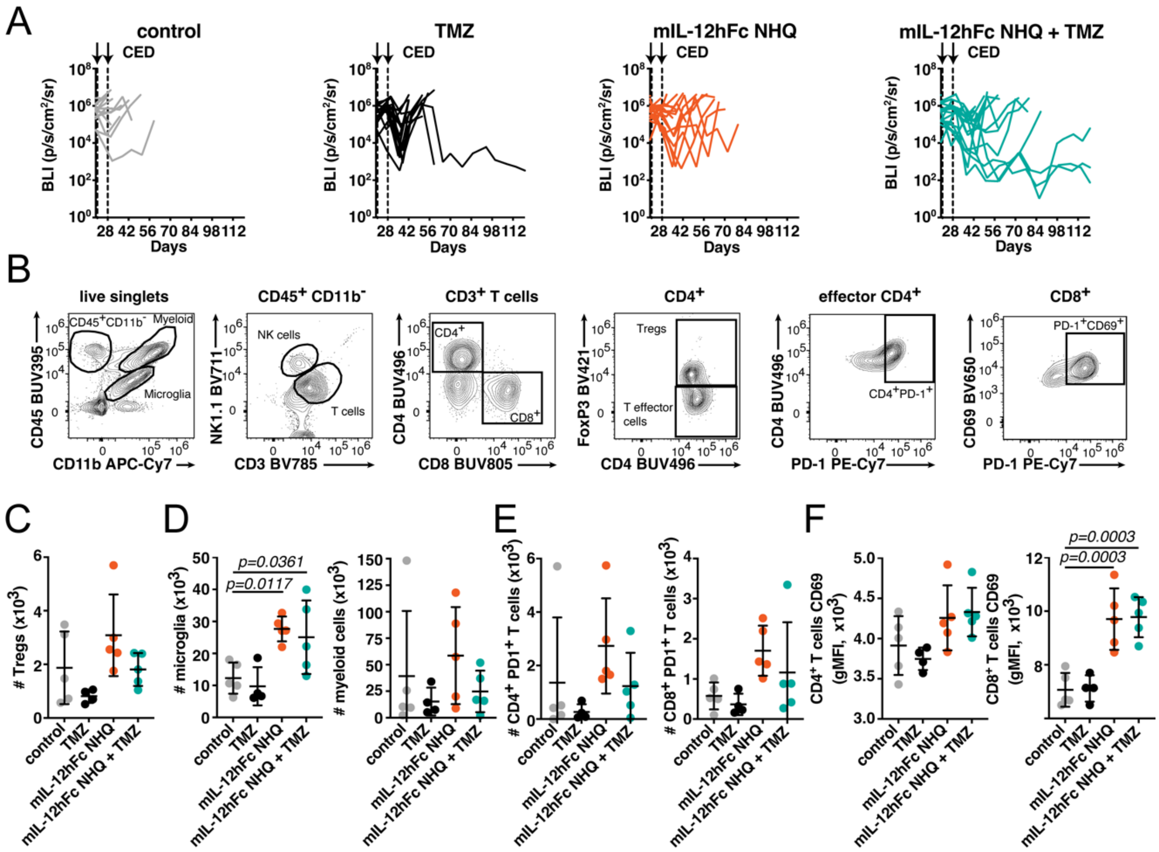

Figure S7. mIL-12hFc NHQ synergizes with TMZ chemotherapy and triggers a locally sustained immune response. (A) Bioluminescence (BLI) traces per animal from experiment described in Figure 5B. **(B)** Gating strategy for GL-261 tumor-infiltrating lymphocyte (TIL) analysis, described in Figure 5 D-G. Live single cells were gated on CD45 and CD11b to define myeloid infiltrating cells, microglia and CD45$^+$ CD11b$^-$ cells. From the CD45$^+$ CD11b$^-$ population, NK cells and CD3$^+$ T cells were identified. CD3$^+$ T cells were further divided into CD4$^+$ and CD8$^+$ T cells. Within the CD4$^+$ T cell population, effector CD4$^+$ T cells and regulatory T cells (Tregs) were distinguished based on FoxP3 expression. Effector CD4$^+$ and CD8$^+$ T cells were then further evaluated for PD1, Ki67 and CD69 expression. Similarly, myeloid infiltrates and microglia were evaluated based on MHCII expression. **(C-F)** Quantitative analysis of populations gated in B. One-way ANOVA with Dunnett's test to the control groups. Only statistically significant differences are shown. Mean ± SD. Control: n=5, TMZ: n=4, mIL-12hFc NHQ: n=5, mIL-12hFc NHQ + TMZ: n=5 animals. Total number of **(C)** Tregs and **(D)** microglia and myeloid infiltrates in the tumor-bearing hemisphere. **(E)** Total number of CD4$^+$ PD1$^+$ and CD8$^+$ PD1$^+$ T cells as well as **(F)** gMFI of CD69 on CD4$^+$ T effector and total CD8$^+$ T cells in the tumor-bearing hemisphere. Source data are provided as a Source Data file.

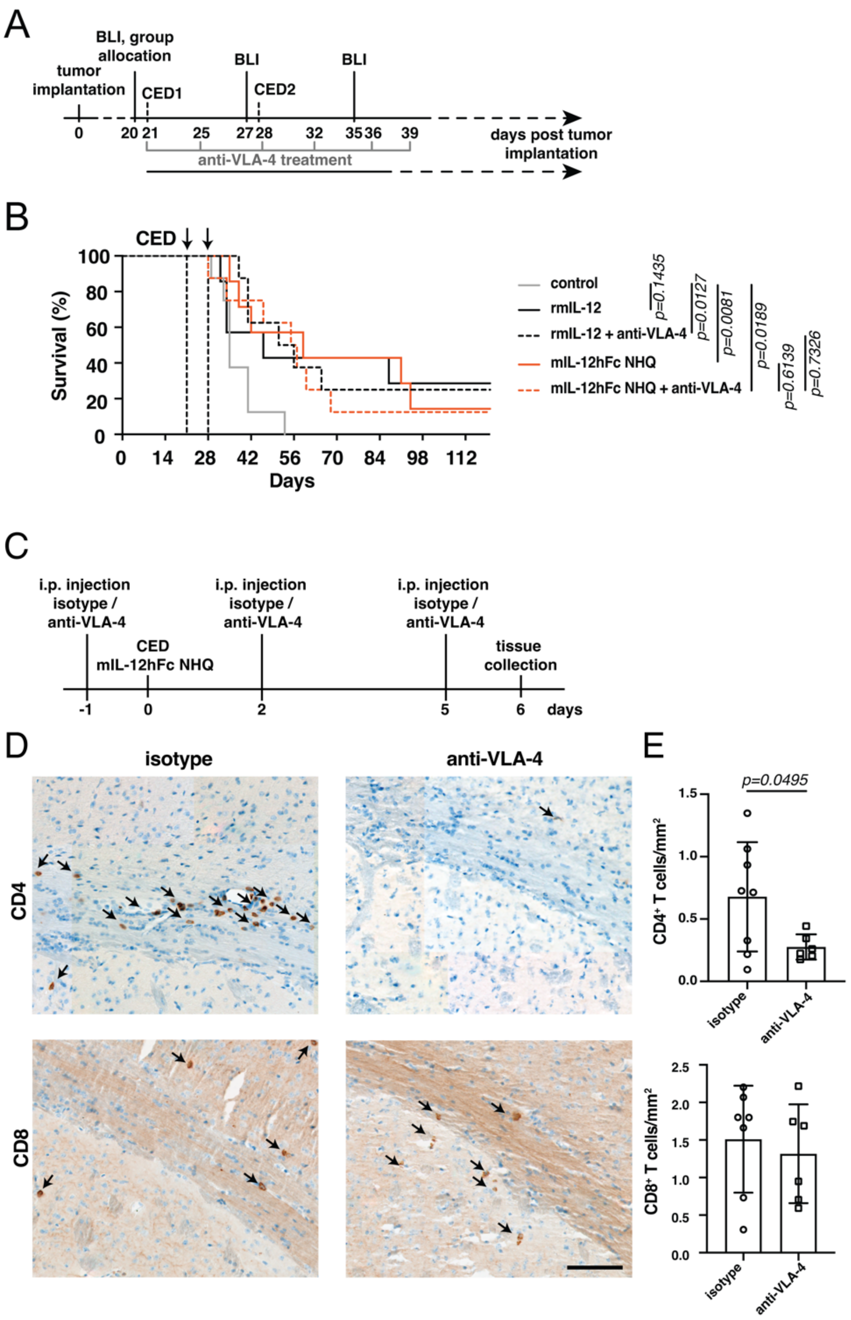

Figure S8. Inhibition of peripheral T cell recruitment (A) GL-261 brain-tumor-bearing animals were treated as in Figure 3, but with equimolar amounts (adjusted to IL-12 stoichiometry) of 1 µg of mIL-12hFc NHQ or 0.67 µg rmIL-12. 100 µg of anti-VLA-4 antibody or its corresponding isotype control were injected twice a week intraperitoneally (i.p). **(B)** Kaplan-Meyer curves of mice described in A) treated with buffer control (gray), rmIL-12 + isotype control (black, solid line), rmIL-12 + anti-VLA-4 antibody (black, dashed line), mIL-12hFc NHQ + isotype control (orange, solid line) or mIL-12hFc NHQ + anti-VLA-4 antibody (orange, dashed line). Log-rank (Mantel-Cox) test compared to controls. n=8 animals/group. **(C)** WT tumor-naïve animals were subjected to three i.p. injections of anti-VLA-4 antibody or its corresponding isotype control, with the first dose given a day prior to intracerebral injection of 1 µg mIL-12hFc NHQ via CED. 24h after the last i.p. injection, brain tissues were collected. **(D, E)** Immunohistochemical quantification of CD4^{+} and CD8^{+} T cells. Data are represented as cell numbers per mm^{2}. mIL-12hFc NHQ + isotype: n=8, mIL-12hFc NHQ + anti-VLA-

4: n=6 animals/group. Unpaired two-tailed t-test. Mean ± SD. Scale bar in D) 100 µm. Source data are provided as a Source Data file.

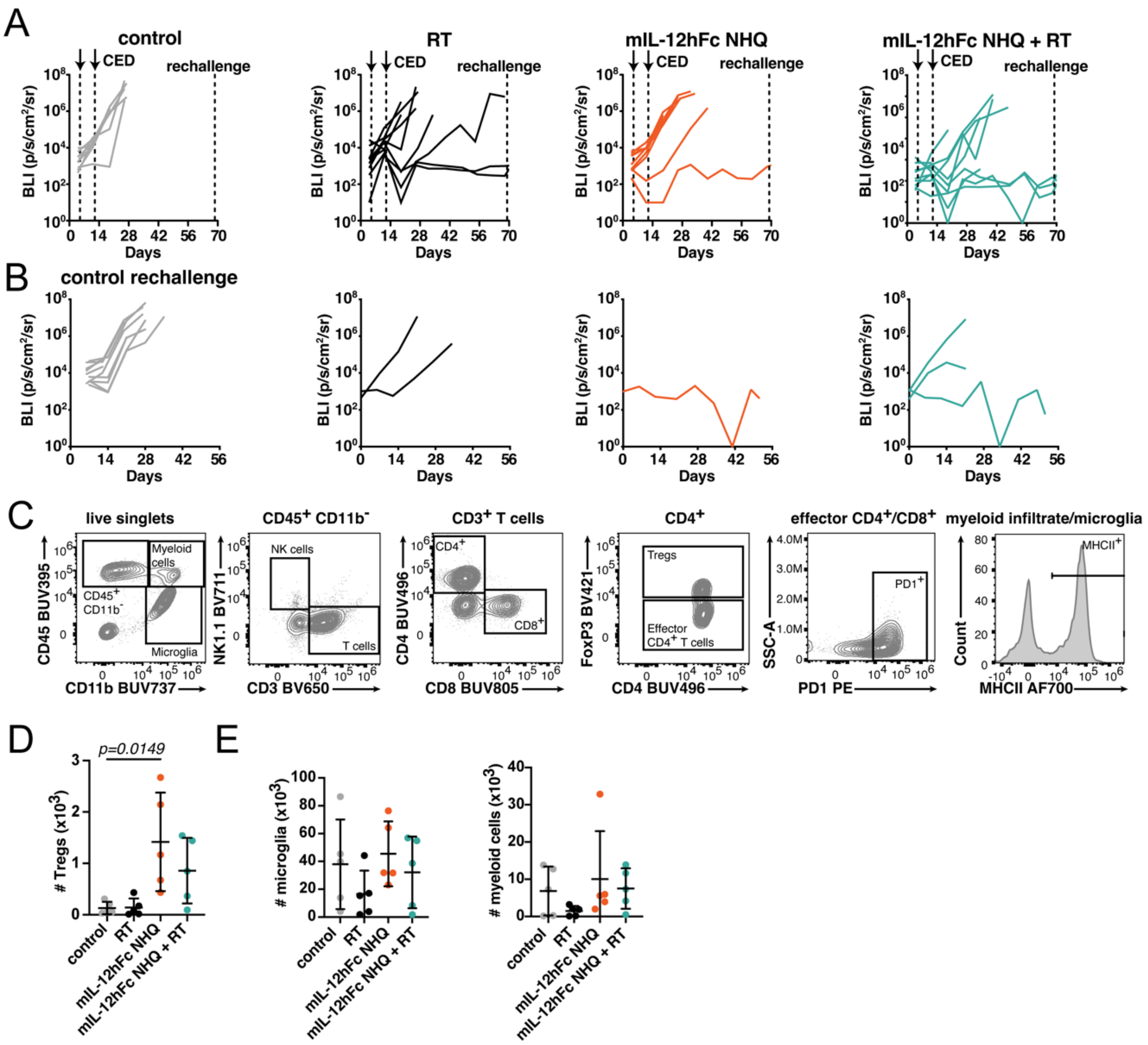

Figure S9. mIL-12hFc NHQ confers partial protection against rechallenge in contralateral hemisphere. Animals from experiments described in Figure 5H that exhibited durable and complete treatment response as indicated by at least three consecutive baseline BLI readings were rechallenged by a second implantation of SB28 cells on day 69 in the left, contralateral hemisphere, at equal coordinates. In parallel, ten C57BL/6 tumor-naïve WT animals received implants in the left striatum as controls. n=9 animals/group. Individual bioluminescence (BLI) traces/animal, **(A)** depicted according to initial treatment for days 0-70 and **(B)** after tumor rechallenge (day 1-51 from the day of rechallenge/day 69-120 from the initial tumor injection, respectively). Control rechallenge: n=10, RT: n=2, mIL-12hFc NHQ: n=1, mIL-12hFc NHQ + RT: n=3 animals. **(C)** Gating strategy for SB28 TIL analysis. Live single cells were gated on CD45 and CD11b to define myeloid infiltrating cells, microglia and CD45$^+$ CD11b$^-$ cells. From the CD45$^+$ CD11b$^-$ population, NK cells and CD3$^+$ T cells were identified. CD3$^+$ T cells were further divided into CD4$^+$ and CD8$^+$ T cells. Within the CD4$^+$ T cell population, Effector CD4$^+$ T cells and regulatory T cells (Tregs) were distinguished based on FoxP3 expression. Effector CD4$^+$ and CD8$^+$ T cells were further evaluated for PD1 expression. Myeloid infiltrates and microglia were evaluated for MHCII expression. Total number of **(D)** Tregs or **(E)** microglia and myeloid infiltrates in the tumor-bearing hemisphere. One-way ANOVA with Dunnett's test to control groups. Only statistically significant differences are shown. Mean ± SD. n=5 animals/group. Source data are provided as a Source Data file.

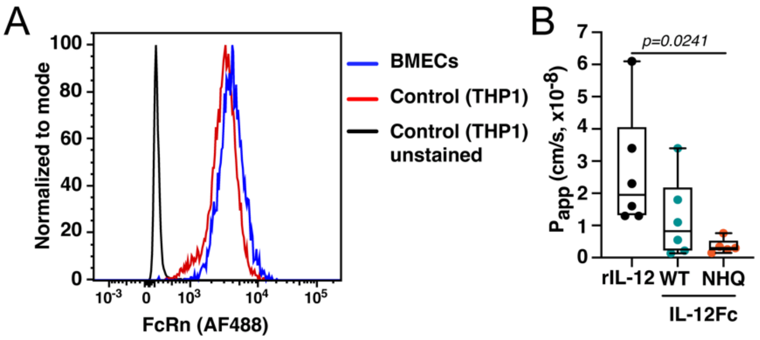

Figure S10. FcRn silenced IL-12Fc exhibits substantially lower transport rates over a human *in vitro* BBB as compared to rIL-12. (A) Flow cytometric analysis of FcRn expression on iPSC derived brain microvascular endothelial cells (BMECs) as compared to a control FcRn-expressing cell line (THP1 [88]). Unstained THP1 control (black), stained THP1 cells (red), stained BMECs (blue). **(B)** Transport rates of IL-12 variants across a BMEC monolayer in the apical (blood) to basolateral (brain) direction. One-way ANOVA with Dunnett's test to the IL-12Fc NHQ group. Whiskers represents the minimum to maximum, middle line represents the mean. Outlier removal based on Grubbs' test. rIL-12 n=6, IL-12Fc WT: n=6, IL-12Fc NHQ: n=5 experiment repetitions. Source data are provided as a Source Data file.

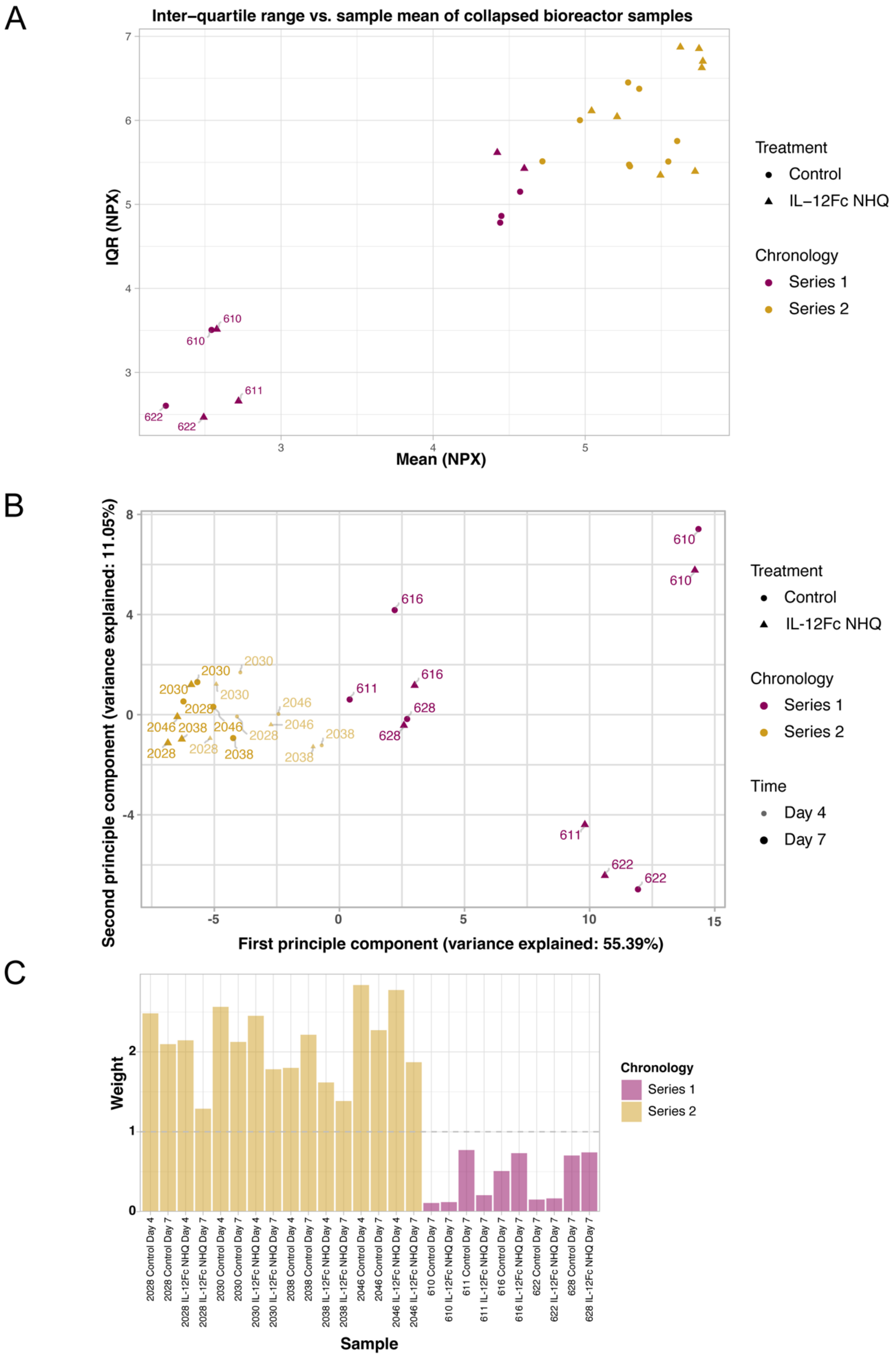

A
Inter−quartile range vs. sample mean of collapsed bioreactor samples
IQR (NPX)
Mean (NPX)
610
610
611
622
622
Treatment
Control
IL−12Fc NHQ
Chronology
Series 1
Series 2
B
Second principle component (variance explained: 11.05%)
First principle component (variance explained: 55.39%)
610
610
616
616
611
628
628
611
622
622
2030
2030
2030
2030
2028
2028
2028
2028
2046
2046
2046
2046
2038
2038
2038
2038
Treatment
Control
IL-12Fc NHQ
Chronology
Series 1
Series 2
Time
Day 4
Day 7
C
Weight
Sample
Chronology
Series 1
Series 2
2028 Control Day 4
2028 Control Day 7
2028 IL-12Fc NHQ Day 4
2028 IL-12Fc NHQ Day 7
2030 Control Day 4
2030 Control Day 7
2030 IL-12Fc NHQ Day 4
2030 IL-12Fc NHQ Day 7
2038 Control Day 4
2038 Control Day 7
2038 IL-12Fc NHQ Day 4
2038 IL-12Fc NHQ Day 7
2046 Control Day 4
2046 Control Day 7
2046 IL-12Fc NHQ Day 4
2046 IL-12Fc NHQ Day 7
610 Control Day 7
610 IL-12Fc NHQ Day 7
611 Control Day 7
611 IL-12Fc NHQ Day 7
616 Control Day 7
616 IL-12Fc NHQ Day 7
622 Control Day 7
622 IL-12Fc NHQ Day 7
628 Control Day 7
628 IL-12Fc NHQ Day 7

Figure S11. Sample weight adjustment based on sample quality decreases uncertainty in the human explant proteomics analysis. Samples in Series 1 had lower overall signal intensity (one biological replicate; patients 610, 611, 616, 622, 628) than Series 2 (3-4 biological replicates - parallel reactors/treatment; patients 2028, 2030, 2038, 2046). In order to increase model precision while retaining all biological information, samples with technical replicates were averaged and regression weights were adjusted to reflect sample assay quality. **(A)** Mean vs. inter-quartile range (IQR) plot, of normalized protein expression (NPX), identifying low signal strength outliers. All technical replicates were collapsed. **(B)** Principle component analysis (PCA) plot of the first two principal components (w/o bioreactor medium control value). **(C)** Estimated quality weights used in the linear model. The timepoints for series 2 were collapsed for further analysis. Outliers as shown in (A) are taken into account with empirical array quality weights. Source data are provided as a Source Data file.

Table S1. Screened variants of IL-12Fc and their stability after protein G affinity purification.

Name	Position 253	Position 310	Position 435	Retained affinity to Protein G	Retained affinity to Protein A	EC_{50} on reporter cell line (ng/ml)	
						Average	SD
**WT (IHH)**	I	H	H	+	+	2.29	±0.00
**AAA** [24, 25]	A	A	A	+	-	2.12	±0.06
**AAE**	A	A	E	+	-	2.21	±0.06
**AAH**	A	A	H	+	-	1.86	±0.26
**AAQ**	A	A	Q	+	-	2.18	±0.30
**AHH**	A	H	H	+	-	2.48	±0.58
**AHQ**	A	H	Q	+	-	2.52	±0.23
**IAQ** [27]	I	A	Q	+	-	2.48	±0.59
**NAA**	N	A	A	-	-	1.21	±0.81
**NAE**	N	A	E	-	-	2.77	±0.05
**NAH**	N	A	H	-	-	2.77	±0.46
**NAQ**	N	A	Q	-	-	5.45	±0.07
**NHH**	N	H	H	+	-	2.29	±0.20
**NHQ**	N	H	Q	+	-	2.01	±0.06

Table S2. Plasma and brain concentrations of IL-12, IL-12Fc WT and IL-12Fc NHQ.

	Treatment:	**IL-12**	**IL-12Fc WT**	**IL-12Fc NHQ**
Organ:	Time point:	Concentration (± SD):		
Brain	10 minutes	3565 ± 812 pg/mg of brain tissue (n=7)	3798 ± 1314 pg/mg of brain tissue (n=8)	4685 ± 1079 pg/mg of brain tissue (n=7)
	6 hours	2248 ± 448 pg/mg of brain tissue (n=7)	2838 ± 602 pg/mg of brain tissue (n=9)	3180 ± 621 pg/mg of brain tissue (n=9)
	1 day	1667 ± 586 pg/mg of brain tissue (n=7)	2147 ± 631 pg/mg of brain tissue (n=9)	2670 ± 1051 pg/mg of brain tissue (n=9)
	3 days	540 ± 318 pg/mg of brain tissue (n=7)	994 ± 856 pg/mg of brain tissue (n=7)	770 ± 579 pg/mg of brain tissue (n=7)
	7 days	27 ± 15 pg/mg of brain tissue (n=8)	233 ± 149 pg/mg of brain tissue (n=7)	122 ± 57 pg/mg of brain tissue (n=8)
Plasma	10 minutes	332 ± 437 pg/mL (n=4)	201 ± 289 pg/mL (n=6)	210 ± 190 pg/mL (n=6)
	6 hours	13035 ± 9118 pg/mL (n=7)	1348 ± 995 pg/mL (n=9)	926 ± 356 pg/mL (n=7)
	1 day	2405 ± 584 pg/mL (n=7)	796 ± 172 pg/mL (n=9)	405 ± 283 pg/mL (n=8)
	3 days	828 ± 611 pg/mL (n=7)	236 ± 174 pg/mL (n=7)	171 ± 133 pg/mL (n=6)
	7 days	36 ± 20 pg/mL (n=8)	94 ± 91 pg/mL (n=7)	64 ± 70 pg/mL (n=8)

Group size in brackets. Outlier removal based on Grubbs' test. Source data are provided as a Source Data file.

Table S3. Patient information.

Identifier	Sex	Series	Number of bioreactors	Age at diagnosis	Diagnosis/Pathology	Pre-treatment	IDH status	MGMT promoter methylation
610	Female	1	1	69	Glioblastoma, Grade IV	None	Wild type	Yes
611	Male	1	1	78	Glioblastoma, Grade IV	None	Wild type	No
616	Female	1	1	*no consent for data sharing*				
622	Female	1	1	49	Grade III glioma	Levetiracetam	Mutant	No
628	Male	1	1	58	Glioblastoma, Grade IV	Steroids	Wild type	Yes
2028	Female	2	3	39	Glioblastoma (methylation class RTK II)	Levetiracetam	Wild type	Yes
2030	Male	2	3	71	Glioblastoma (methylation class RTK II)	None	Wild type	Yes
2038	Male	2	4	92	Glioblastoma	Levetiracetam	Wild type	Yes
2046	Male	2	3	61	Diffuse type high-grade glioma	None	N/A	Yes

Supplemental References

20. Buser DP*, et al.* Quantitative proteomics reveals reduction of endocytic machinery components in gliomas. *EBioMedicine* **46**, 32-41 (2019).

24. Medesan C, Matesoi D, Radu C, Ghetie V, Ward ES. Delineation of the amino acid residues involved in transcytosis and catabolism of mouse IgG1. *J Immunol* **158**, 2211-2217 (1997).

25. Kim JK, Firan M, Radu CG, Kim CH, Ghetie V, Ward ES. Mapping the site on human IgG for binding of the MHC class I-related receptor, FcRn. *Eur J Immunol* **29**, 2819-2825 (1999).

27. Kenanova V*, et al.* Tailoring the pharmacokinetics and positron emission tomography imaging properties of anti-carcinoembryonic antigen single-chain Fv-Fc antibody fragments. *Cancer research* **65**, 622-631 (2005).

84. Uhlen M*, et al.* A pathology atlas of the human cancer transcriptome. *Science* **357**, (2017).

85. Puchalski RB*, et al.* An anatomic transcriptional atlas of human glioblastoma. *Science* **360**, 660-663 (2018).

86. Sellers RS*, et al.* Society of Toxicologic Pathology position paper: organ weight recommendations for toxicology studies. *Toxicol Pathol* **35**, 751-755 (2007).

87. Queck A*, et al.* Systemic MCP-1 Levels Derive Mainly From Injured Liver and Are Associated With Complications in Cirrhosis. *Front Immunol* **11**, 354 (2020).

88. Zhu X*, et al.* MHC class I-related neonatal Fc receptor for IgG is functionally expressed in monocytes, intestinal macrophages, and dendritic cells. *J Immunol* **166**, 3266-3276 (2001).
