# Supplementary material for: FcRn-silencing of IL-12Fc prevents toxicity of local IL-12 therapy and prolongs survival in experimental glioblastoma
Source: Nat Commun. 2025 May 22;16:4751. doi: 10.1038/s41467-025-59971-0 (PMC12098678; doi:10.1038/s41467-025-59971-0)
Supplement: Supplementary file 3 — Reporting Summary [file 41467_2025_59971_MOESM3_ESM.pdf]

nature portfolio

Corresponding author(s): Johannes vom Berg, johannes.vomberg@uzh.ch

Last updated by author(s): May 8, 2025

Reporting Summary

Nature Portfolio wishes to improve the reproducibility of the work that we publish. This form provides structure for consistency and transparency in reporting. For further information on Nature Portfolio policies, see our Editorial Policies and the Editorial Policy Checklist.

Statistics

For all statistical analyses, confirm that the following items are present in the figure legend, table legend, main text, or Methods section.

n/a	Confirmed	
☐	☒	The exact sample size (*n*) for each experimental group/condition, given as a discrete number and unit of measurement
☐	☒	A statement on whether measurements were taken from distinct samples or whether the same sample was measured repeatedly
☐	☒	The statistical test(s) used AND whether they are one- or two-sided<br>*Only common tests should be described solely by name; describe more complex techniques in the Methods section.*
☐	☒	A description of all covariates tested
☐	☒	A description of any assumptions or corrections, such as tests of normality and adjustment for multiple comparisons
☐	☒	A full description of the statistical parameters including central tendency (e.g. means) or other basic estimates (e.g. regression coefficient) AND variation (e.g. standard deviation) or associated estimates of uncertainty (e.g. confidence intervals)
☐	☒	For null hypothesis testing, the test statistic (e.g. *F*, *t*, *r*) with confidence intervals, effect sizes, degrees of freedom and *P* value noted<br>*Give P values as exact values whenever suitable.*
☒	☐	For Bayesian analysis, information on the choice of priors and Markov chain Monte Carlo settings
☒	☐	For hierarchical and complex designs, identification of the appropriate level for tests and full reporting of outcomes
☐	☒	Estimates of effect sizes (e.g. Cohen's *d*, Pearson's *r*), indicating how they were calculated

Our web collection on statistics for biologists contains articles on many of the points above.

Software and code

Policy information about availability of computer code

Data collection

Data collection for this study involved the acquisition of various datasets, including in vivo data from different mouse strains, clinical data from human participants and experimental data from biological assays. All data collection procedures were conducted following approved ethical protocols and standard operating procedures.

Data analysis

Data analysis for this study was performed using a combination of statistical methods and specialized software tools. For all non-survival analyses involving two experimental groups, a two-tailed Student's t test was conducted. When comparing three or more experimental groups, a one-way ANOVA with Šídák's correction for multiple testing was applied. Kaplan-Meier survival curves were analyzed using the Log-rank (Mantel-Cox) test to determine statistical significance. P-values less than 0.05 were considered statistically significant and are indicated in the respective figures.
All quantitative analyses were conducted using MS Excel (Microsoft) and GraphPad Prism version 10.2.3 for Mac OSX (GraphPad Software, Inc). Flow cytometry data were analyzed using FlowJo Software v10 (BD). No custom code or software was developed or used in this study. Detailed methods and tools used for data analysis are provided in the Methods section of the manuscript.

For the proteomics dataset, data was analyzed in line with recommendations by Olink and as described in the methods section of the manuscript, including references therein. For exploratory analysis, the data were scaled and centered to z-scores. The principal components of the transformed counts were calculated using the prcomp function of the stats package version 4.3.1, written in the statistical computing software R. Differential expression analysis was performed with the linear regression tools from the R package limma version 3.56.2. The differential expression statistics were estimated with two-sided, moderated t-tests. The linear model included the additive factors Patient, Treatment and Time. The function limma::arrayWeights was used for evaluating quality weights of the individual samples. The regression details include a robust hyperparameter estimation and a calculation of the protein mean–variance trend. The gene set analysis focuses on the hallmark gene sets defined for the Molecular Signatures Database of the Broad Institute. These gene sets were retrieved with the msigdbr

package version 7.5.1 on 2023-11-28. Statistical relevance was determined by rotational gene set tests implemented in the limma::mroast function. The rotational tests incorporated the same design formula as in the linear model of the protein expressions. The quality weights were carried over as well. False discovery rate (FDR) adjustment was accomplished using the Benjamini–Hochberg method.
The version of the R computing language is 4.3.1 (2023-06-16).

For manuscripts utilizing custom algorithms or software that are central to the research but not yet described in published literature, software must be made available to editors and reviewers. We strongly encourage code deposition in a community repository (e.g. GitHub). See the Nature Portfolio guidelines for submitting code & software for further information.

Data

Policy information about availability of data

All manuscripts must include a data availability statement. This statement should provide the following information, where applicable:
- Accession codes, unique identifiers, or web links for publicly available datasets
- A description of any restrictions on data availability
- For clinical datasets or third party data, please ensure that the statement adheres to our policy

The flow cytometry data generated in this study have been deposited in the Zenodo database under accession code 15263559 [doi:10.5281/zenodo.15263559, https://zenodo.org/records/15263560]. The huma proteomics data generated in this study have been deposited in the Zenodo database under accession code 15282130 [doi:10.5281/zenodo.15282129, https://zenodo.org/records/15282130]. All other data generated in this study are provided in the supplementary information file and source data file.

Research involving human participants, their data, or biological material

Policy information about studies with human participants or human data. See also policy information about sex, gender (identity/presentation), and sexual orientation and race, ethnicity and racism.

Reporting on sex and gender

The study involved human brain tumor explant samples from patients diagnosed with glioblastoma. The data collected included the sex of each participant, which was recorded and used in the analysis of treatment effects on the explants. However, the study did not specifically analyze differences in treatment effects based on sex or gender due to the limited sample size. Sex-specific data is available but was not a primary focus of this study.

Reporting on race, ethnicity, or other socially relevant groupings

The participants' race, ethnicity, or other socially relevant groupings were not recorded as part of this study. The research focused on the biological characteristics of glioblastoma tumors and their response to treatment, rather than on any socio-demographic variables. As such, no analysis or conclusions were drawn regarding race or ethnicity.

Population characteristics

The study utilized human glioblastoma explants obtained from a group of patients with the following characteristics:
-Sex: Both male and female patients were included in the study.
-Age at Diagnosis: Information on the age at diagnosis was recorded for all participants.
-Diagnosis/Pathology: All patients were diagnosed with glioblastoma / high grade glioma.
-Pre-treatment: Data on prior treatments received by the patients were collected.
-IDH Status: The isocitrate dehydrogenase (IDH) mutation status was determined for each patient.
-MGMT Promoter Methylation: The methylation status of the O6-methylguanine-DNA methyltransferase (MGMT) promoter was also assessed.
The study also included an ex vivo blood-brain barrier (BBB) assay using induced pluripotent stem cells (iPSCs) generated from human dermal skin fibroblasts obtained from a healthy adult donor. The donor was not part of any specific patient population, selected solely for the derivation of the iPSC lines.

Recruitment

For the glioblastoma patient explant study, participants were recruited from University Hospital Basel, Basel, Switzerland. Recruitment was based on clinical need for surgery and the availability of excess tumor tissue for research purposes. Patients provided informed consent prior to participation in the study.

For the ex vivo BBB assay, dermal fibroblasts were obtained from a single healthy donor who provided informed consent. This donor was recruited without consideration of socio-demographic characteristics, and the iPSC line was derived for research purposes only.

Ethics oversight

The study received ethics approval from the following committees:
1. Human Brain Tumor Explants: The research involving glioblastoma explants was approved by the Ethics Committee of University Hospital Basel, Basel, Switzerland (ethics approval number: Req-2019-00553). Informed consent was obtained from all participants prior to sample collection and participation.
2.Ex vivo BBB Assay: The ex vivo blood-brain barrier assay was approved by the Ethics Committee of Zurich University of Applied Sciences, Wädenswil, Switzerland (ethics approval number: KEK-ZH-Nr: 2015-0589). The iPSC cell line was generated from dermal fibroblasts obtained from a healthy donor, who provided informed consent.

Note that full information on the approval of the study protocol must also be provided in the manuscript.

Field-specific reporting

Please select the one below that is the best fit for your research. If you are not sure, read the appropriate sections before making your selection.

☒ Life sciences ☐ Behavioural & social sciences ☐ Ecological, evolutionary & environmental sciences

For a reference copy of the document with all sections, see nature.com/documents/nr-reporting-summary-flat.pdf

Life sciences study design

All studies must disclose on these points even when the disclosure is negative.

Sample size	The sample size for each experiment was determined based on previous studies and the expected effect size. For every experiment, number of animals used were specific in each figure legend.
Data exclusions	Data were included in the analysis if they met the predefined criteria for quality and completeness. Any samples or data points that were technically compromised due to processing errors, contamination or incomplete data were excluded from the analysis. All exclusions were made before the data analysis and were not influenced by experimental outcomes.
Replication	All experiments were performed with sufficient biological and technical replicates to ensure reliability and reproducibility of the results. Consistent results across replicates demonstrate the robustness of our findings.
Randomization	Where applicable, randomization was employed to assign samples to different experimental groups. Such as, all survival and brain retention experiment animals were randomly assigned to treatment groups to minimize bias. Randomization procedures for tumor experiments were performed based on the BLI data obtained from in vivo imaging to ensure unbiased distribution across groups.
Blinding	Blinding was implemented where feasible to minimize bias in data collection and analysis. For all in vivo experiments and patient explant experiments, researchers conducting the treatments and those analyzing the outcomes were blinded to the group assignments. For flow cytometry and other data analyses, blinding was maintained by using coded sample identifiers, so that the analysts were unaware of the treatment groups during data analysis. Blinding was not applicable in some experiments where visual identification of experimental conditions was unavoidable, but data analysis remained blinded.

Reporting for specific materials, systems and methods

We require information from authors about some types of materials, experimental systems and methods used in many studies. Here, indicate whether each material, system or method listed is relevant to your study. If you are not sure if a list item applies to your research, read the appropriate section before selecting a response.

Materials & experimental systems

n/a	Involved in the study
☐	☒ Antibodies
☐	☒ Eukaryotic cell lines
☒	☐ Palaeontology and archaeology
☐	☒ Animals and other organisms
☐	☒ Clinical data
☒	☐ Dual use research of concern
☒	☐ Plants

Methods

n/a	Involved in the study
☒	☐ ChIP-seq
☐	☒ Flow cytometry
☒	☐ MRI-based neuroimaging

Antibodies

Antibodies used

Flow cytometry antibodies used in the study
(Marker, Fluorophore, Host and isotype, Clone, Manufacturer, Catalogue number, Lot, Dilution)
CD3 AF700 Rat IgG2b, κ 17A2 Biolegend 100215 B197779 1:100
CD3 BV650 Armenian hamster IgG1, κ 145-2C11 BD Bioscience 564378 3019447 1:50
CD3 BV785 Armenian hamster IgG 145-2C11 Biolegend 100355 B325355 1:100
CD4 PerCP-Cy5.5 Rat IgG2b, κ GK1.5 Biolegend 100433 B193201 1:200
CD4 BUV496 Rat IgG2b, κ GK1.5 BD Biosciences 612952 3082211 1:200
CD8α Super Bright 780 Rat IgG2a, κ 53-6.7 Biolegend 100750 B239317 1:400
CD8α BV785 Rat IgG2a, κ 53-6.7 Biolegend 100750 B239317 1:400
CD8α BUV805 Rat IgG2a, κ 53-6.7 BD Biosciences 612898 3317931 1:200
CD8α FITC Rat IgG2a, κ 53-6.7 Biolegend 100706 B240642 1:200
CD11b PE Rat IgG2b, κ M1/70 Biolegend 101207 B181097 1:200
CD11b BV711 Rat IgG2b, κ M1/70 Biolegend 101242 B235643 1:800
CD11b BUV737 Rat IgG2b, κ M1/70 BD Bioscience 563015 2325988 1:200
CD11b APC-Cy7 Rat IgG2b, κ M1/70 Biolegend 101226 B238268 1:200
CD11c APC Armenian hamster IgG N418 Biolegend 117309 B262129 1:400
CD11c BV785 Armenian hamster IgG N418 Biolegend 117335 B384994 1:100
CD19 Pacific Blue Rat IgG2a, κ 6D5 Biolegend 115523 B203467 1:100
CD25 PE-Cy7 Rat IgG1, λ PC61 Biolegend 102045 B274184 1:100
CD44 FITC Rat IgG2b, κ IM7 Biolegend 103006 B147985 1:200
CD45 APC-Cy7 Rat IgG2b, κ 30-F11 Biolegend 103115 B181872 1:200

CD45 BUV395 Rat IgG2b, κ 30-F11 BD Biosciences 564279 3205129 1:400
CD45 BUV395 Rat IgG2b, κ 30-F11 BD Bioscience 564279 2259805 1:400
CD62L PE/Dazzle 594 Rat IgG2a, κ MEL-14 Biolegend 104447 B238771 1:400
CD64 BV605 Mouse IgG1, κ x54-5/7.1 Biolegend 139323 B240410 1:400
CD69 BV650 Armenian hamster IgG H1.2F3 Biolegend 104541 B245647 1:400
CD103 PE Armenian hamster IgG 2E7 Biolegend 121405 B164141 1:200
CD103 APC Armenian hamster IgG 2E7 ThermoFisher 17-1031-82 E07197-1631 1:100
CD127 BUV737 Rat IgG2b, κ SB199 BD Biosciences 612841 3251773 1:100
CD137 (4-1BB) PE Syrian hamster IgG 17B5 Biolegend 106106 B418365 1:200
CD206 BV650 Rat IgG2a, κ C068C2 Biolegend 141723 B313883 1:200
CD223 (Lag3) PE Rat IgG1, κ C9B7W Biolegend 125207 B290395 1:200
CD274 (PD-L1) PE/Dazzle 594 Rat IgG2b, κ 10F.9G2 Biolegend 124324 B256601 1:100
CD279 (PD-1) PE-Cy7 Rat IgG2a, κ 29F.1A12 Biolegend 135215 B202870 1:100
F4/80 FITC Rat IgG2a, κ BM8 Biolegend 123107 B177256 1:100
F4/80 PerCP-Cy5.5 Rat IgG2a, κ BM8 Biolegend 123126 B198161 1:100
FoxP3 PE Mouse IgG1, κ 150D Biolegend 320008 B275698 1:100
FoxP3 BV421 Rat IgG2b, κ MF23 BD Biosciences 562996 2251201 1:200
Granzyme B FITC Mouse IgG1, κ QA16A02 Biolegend 372205 B274305 1:100
Granzyme B BV510 Mouse IgG1, κ GB11 BD Biosciences 563388 3317967 1:50
IFNγ APC Mouse IgG1, κ XMG1.2 Biolegend 505809 B248690 1:100
Ki67 BV605 Rat IgG2b, κ 16A8 Biolegend 652413 B242902 1:400
KLRG1 PE/Dazzle 594 Syrian hamster IgG 2F1 Biolegend 138423 B364422 1:200
Ly-6C PerCP-Cy5.5 Rat IgG2c, κ HK1.4 Biolegend 128011 B250461 1:400
Ly-6C BUV563 Rat IgG1, κ HK1.4.rMAb BD Bioscience 755198 4173495 1:100
Ly-6G AF700 Rat IgG2a, κ 1a8 Biolegend 127621 B266370 1:100
Ly-6G BUV615 Rat IgG2a, κ 1A8 BD Bioscience 751263 4173484 1:100
MHC-II Pacific Blue Mouse IgG2a, κ AF6-120.1 Biolegend 116421 B240824 1:400
MHC-II AF700 Rat IgG2b, κ M5/144.15.2 Biolegend 107621 B264455 1:100
NK1.1 PE-Cy7 Mouse IgG2a, κ pk136 Biolegend 108714 B212623 1:400
NK1.1 BV605 Mouse IgG2a, κ pk136 Biolegend 108739 B261612 1:200
NK1.1 BV711 Mouse IgG2a, κ pk136 Biolegend 108745 B251561 1:100
PD-1 PE Rat IgG2b, κ RPM1-30 Biolegend 109104 B367713 1:50
T-bet PerCP Cy5.5 Mouse IgG1, κ 4B10 ThermoFisher 45-5825-80 2018410 1:50
TCRβ PE-Cy7 Armenian hamster IgG H57-597 Biolegend 109222 B241527 1:200
TCRγδ PE-Cy7 Armenian hamster IgG GL3 Biolegend 118124 B172106 1:400
Tim3 APC Rat IgG1, κ 8B.2C12 eBioscience 17-5871-82 1995482 1:200
TNFα Pacific Blue Rat IgG1, κ MP6-XT22 Biolegend 506318 B213609 1:200
TNFα PE/Dazzle 594 Rat IgG1, κ MP6-XT22 Biolegend 506345 B410364 1:100
Live/dead Zombie aqua n/a n/a Biolegend 77143 B324733 1:200
Live/dead Zombie NIR n/a n/a Biolegend 423105 2251201 1:1000
For all experinent an Fc block was used: Biologend, 101320, B276722

Validation

All antibodies used in this study were selected based on their established specificity and performance in flow cytometry applications. Validation of these antibodies was confirmed through the following approaches:
1. Supplier validation: Each antibody was sourced from reputable suppliers (Biolegend or BD Bioscienes) known for rigorous quality control and validation processes. ,The suppliers provided detailed data sheets confirming the specificity, clonality, and appropriate use of each antibody in flow cytometry.
2. Isotype controls: Appropriate isotype control antibodies were used in advance to confirm that the observed staining was specific to the target antigen and not due to non-specific binding. Isotype controls matched the primary antibodies in species and immunoglobulin subtype, ensuring that background staining was minimal.
3. Positive and negative controls: The validation of each antibody was further confirmed using known positive and negative control cell populations. Positive controls: cells known to express the target antigen were used to verify that the antibodies correctly labeled the expected cell populations. Negative controls: cells that do now express the target antigen as well as unstained controls were included to ensure that no non-specific binding occured.
4. Staining patterns: The staining patterns observed with each antibody were consistent with the expected cellular localization and expression profiles as described in the literature.
5. Internal validation: For antibodies used in intracellular staining (e.g. FoxP3, TNFα, IFNγ) the staining protocol included fixation and permeabilization steps that were validated within the lab to ensure that intracellular targets were accurately detected without significant loss of cell viability or marker expression.
6. Serial Dilutionsfor Optimization: To ensure optimal antibody performance, serial dilutions of each antibody were performed during the initial experiments. This process allowed us to determine the optimal concentration that provided clear, specific staining with minimal background. The antibody concentrations used in the study were based on these titration experiments, ensuring that the antibodies worked effectively under experimental conditions. The specific dilutions for each antibody can be provided if required further.

Eukaryotic cell lines

Policy information about cell lines and Sex and Gender in Research

Cell line source(s)	GL-261 cells: These cells were provided by Dr. A. Fontana at the Institute of Experimental Immunology, University of Zürich, Zürich, Switzerland.<br>SB28 cells: These cells were provided by Dr. H. Okada at the University of California, San Francisco, USA.
Authentication	The GL-261 and SB28 cell lines used in this study were obtained directly from reputable academic sources and were well distinguishable according to in vitro and in vitro growth, phenotypic appearance and expression of luciferase (GL-261) vs luciferase AND GFP (SB28).
Mycoplasma contamination	Both GL-261 and SB28 cell lines were routinely tested for mycoplasma contamination using PCR-based assays and all tests were negative for mycoplasma contamination.
Commonly misidentified lines<br>(See ICLAC register)	The GL-261 and SB28 cell lines are not listed as commonly misidentified cell lines in the International Cell Line Authentication Committee (ICLAC) database. They are well-characterized lines commonly used in glioblastoma research.

Animals and other research organisms

Policy information about studies involving animals; ARRIVE guidelines recommended for reporting animal research, and Sex and Gender in Research

Laboratory animals	C57BL/6J mice: Obtained from Charles River (Sulzfeld, Germany) or Janvier Labs (Le Genest-Saint-Isle, France).<br>B6.Cg-Fcgrttm1Dcr Tg(FCGRT)32Dcr/DcrJ (hFcRn Tg32) mice: Purchased from The Jackson Laboratory (stock number 014565) and bred in house.<br>C3H/HeJ (Heston) mice: Purchased from the Jackson Laboratory (stock number 000659).<br>These laboratory animals were used in the described experimental procedures, and their health and well-being were closely monitored throughout the study.
Wild animals	No wild animals were used in this study.
Reporting on sex	Both male and female mice were used in the experiment, when possible. However, the sex of the animals was not considered in the study design and analysis to account for any potential sex-related differences in outcomes.
Field-collected samples	No field-collected samples were used in this study. All biological materials were obtained from laboratory animals as described
Ethics oversight	All animal procedures were approved by the Cantonal Veterinarian's Office of Zurich under the following licenses: ZH246/2015, ZH194/2019, and ZH175/2022. Ethical considerations included efforts to minimize the number of animals used and their suffering. Humane endpoint criteria were established for tumor and toxicology studies, which included significant body weight loss (>20% over peak weight) or severe clinical symptoms such as hunchback posture, lack of activity, seizures, or loss of motor coordination. Euthanasia was performed using controlled CO2 asphyxiation, in accordance with ethical guidelines.

Note that full information on the approval of the study protocol must also be provided in the manuscript.

Clinical data

Policy information about clinical studies

All manuscripts should comply with the ICMJEguidelines for publication of clinical research and a completedCONSORT checklist must be included with all submissions.

Clinical trial registration	This study did not involve a clinical trial; therefore, no clinical trial registration is applicable.
Study protocol	This study did not involve a clinical trial or the implementation of a clinical study protocol. The use of patient-derived tumor explants was conducted according to established ethical guidelines and approvals, as detailed below.
Data collection	Patient-derived glioblastoma explants were obtained under approved ethical protocols from University Hospital Basel, Basel, Switzerland (Ethics approval Req-2019-00553). Relevant clinical data, including sex, age at diagnosis, diagnosis/pathology, pre-treatment information, IDH status, and MGMT promoter methylation status, were collected retrospectively from patient records and anonymized prior to use in this study
Outcomes	The outcomes measured in this study were related to the treatment effects on patient-derived glioblastoma explants, including assessments of cell viability, molecular markers, and histological changes. These outcomes are described in detail in the Methods and Results sections of the manuscript.

Plants

Seed stocks

Report on the source of all seed stocks or other plant material used. If applicable, state the seed stock centre and catalogue number. If plant specimens were collected from the field, describe the collection location, date and sampling procedures.

Novel plant genotypes

Describe the methods by which all novel plant genotypes were produced. This includes those generated by transgenic approaches, gene editing, chemical/radiation-based mutagenesis and hybridization. For transgenic lines, describe the transformation method, the number of independent lines analyzed and the generation upon which experiments were performed. For gene-edited lines, describe the editor used, the endogenous sequence targeted for editing, the targeting guide RNA sequence (if applicable) and how the editor was applied.

Authentication

Describe any authentication procedures for each seed stock used or novel genotype generated. Describe any experiments used to assess the effect of a mutation and, where applicable, how potential secondary effects (e.g. second site T-DNA insertions, mosiacism, off-target gene editing) were examined.

Flow Cytometry

Plots

Confirm that:

☒ The axis labels state the marker and fluorochrome used (e.g. CD4-FITC).

☒ The axis scales are clearly visible. Include numbers along axes only for bottom left plot of group (a 'group' is an analysis of identical markers).

☒ All plots are contour plots with outliers or pseudocolor plots.

☒ A numerical value for number of cells or percentage (with statistics) is provided.

Methodology

Sample preparation

Immune infiltrate analysis: Tumor-bearing hemispheres from mice were minced and digested in RPMI medium containing 0.2 mg/mL Collagenase D, 0.5 mg/mL DNase I, and 10% FCS. After 30 minutes of incubation at 37°C, the tissue homogenate was filtered through a 70 µm cell strainer, centrifuged, and separated on a Percoll gradient. Cells were then either directly labeled with specific antibodies or restimulated for intracellular cytokine staining. For intracellular labeling, cells were fixed and permeabilized following surface labeling.
FcRn Expression analysis in iPSC-derived BMECs: iPSC-derived BMECs were trypsinized with TrypLE Select, washed, and fixed with 3% PFA. The cells were then permeabilized with 0.1% Saponin before being incubated with AF488-conjugated anti-FcRn antibody in PBS-Saponin buffer for 30 minutes at room temperature.

Instrument

Data acquisition for immune infiltrate analysis was performed using an LSR II Fortessa (BD) or a 5L Cytek Aurora (Cytek Biosciences) flow cytometer. For FcRn expression analysis, data were acquired using a FACS Aria III cytometer (BD).

Software

Flow cytometry data were analyzed using FlowJo Software v10 (BD).

Cell population abundance

Absolute cell counts were determined by adding 5 µL of counting beads (Thermo Fisher Scientific) per sample before data acquisition. The abundance of various cell populations (e.g., CD45+, CD3+, CD4+, CD8a+, CD11b+, etc.) was quantified as a percentage of the total live cell population, which was determined using the Zombie Aqua/NIR live/dead discriminator.

Gating strategy

Immune Infiltrate Analysis:Live cells were identified by gating out dead cells using the Zombie Aqua/NIR discriminator.
Subsequent gates were applied to identify specific immune cell populations based on the expression of surface markers such as CD45, CD3, CD4, CD8a, CD11b, and others. For intracellular cytokine staining, cells were further gated based on the expression of intracellular markers following permeabilization.
FcRn Expression Analysis: iPSC-derived BMECs were first gated for single cells using forward and side scatter, followed by gating on the AF488-positive population to identify FcRn-expressing cells.

☒ Tick this box to confirm that a figure exemplifying the gating strategy is provided in the Supplementary Information.
